# Supplementary material for: Crystallization of Ethylene Plant Hormone Receptor—Screening for Structure
Source: Biomolecules. 2024 Mar 20;14(3):375. doi: 10.3390/biom14030375 (PMC10968091; doi:10.3390/biom14030375)
Supplement: Supplementary file 1 [file biomolecules-14-00375-s001.zip › biomolecules-2888780-supplementary.pdf]

Supplementary Material
for
Crystallization of ethylene plant hormone receptor – screening for the structure

Buket Rüffer [1,¶], Yvonne Thielmann [1,¶], Moritz Lemke [1], Alexander Minges [1] and Georg Groth [1,*]

Author Affiliations:

[1]Heinrich Heine University Düsseldorf, Faculty of Mathematics and Natural Sciences, Institute of Biochemical Plant Physiology, Germany

To whom correspondence should be addressed:

Email: georg.groth@hhu.de

Tel: +49 211 81 12822
Fax: +49 211 81 13569

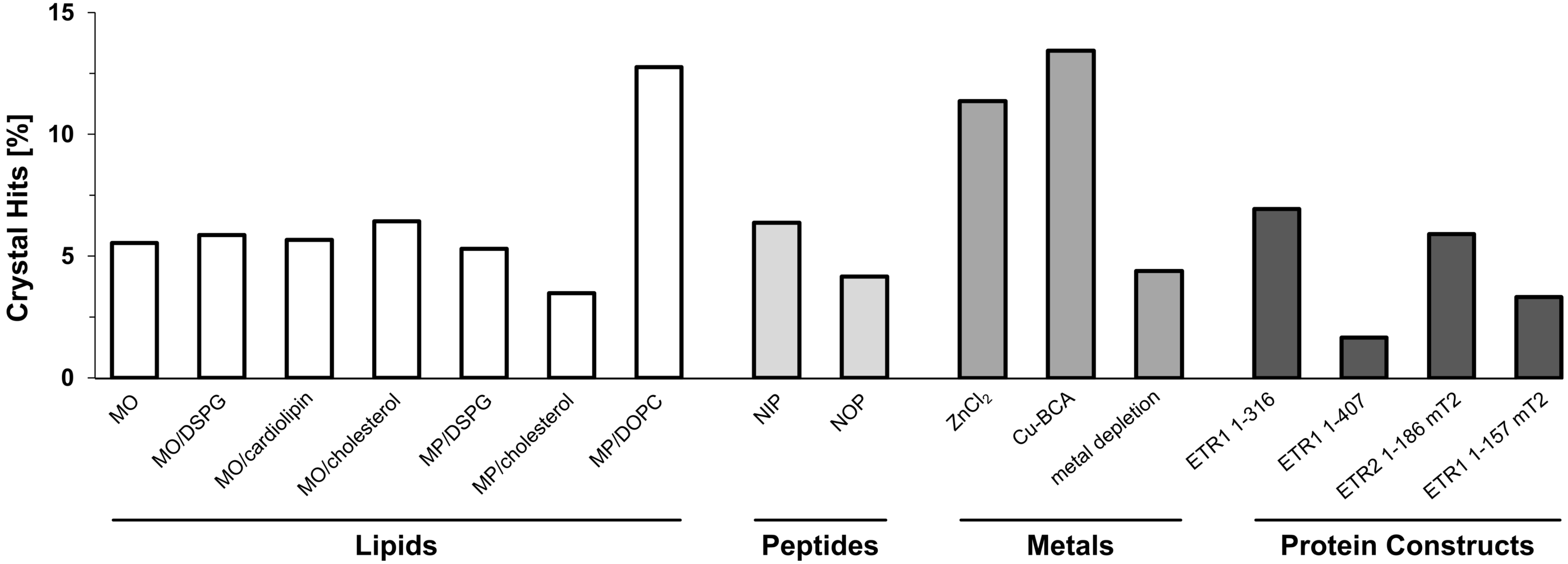

S1 Fig 1. Compared data of crystallization hits of harvested and measured crystals [%], which were evaluated with regard to the following parameters from left to right: lipids (white), peptides (light grey), metals (dark grey) and protein constructs (black).

Table S1. Primer sequences for cloning.

Primer name	Sequence
ETR2 TMD_CFP_for	ATGCTGACCCAGGAAATCCGTAAAAGTCTGATGGTGAGCAAGGGC
ETR2 TMD_CFP_rev	CAGACTTTTACGGATTTCCTG
ETR2 TMD_for	CCGCATCGTGACTGAC
CFP rev	AGGCAGATCGTCAGTCAGTCACGATGCGGCTTGTACAGCTCGTCC
mTurquoise-T65S_for	ACCACCCTGTCCTGGGGC
mTurquoise-T65S rev	CACGAGGGTGGGCCAG
mTurquoise2_A145Y-I146F_for	TACTTTAGCGACAACGTCTATATCACCG
mTurquoise2_A145Y-I146F	GTTGTACTCCAGCTTGTGCC
ETR2_c-term.-10x His-forI	CATCATCATCATCACTGACTGACGATCTGCCTCG
ETR2_CFP_10x His	ATGATGATGATGATGCTTGTACAGCTCGTCCATGC
ETR1 TMD_mT2_for	catgagattagaagcactttaatggtgagcaagggcgaggagctg
GST_TEV_rev	caatacaattgcagacttcCATACCCGGGCCCTGAAAATACAG
ETR1-TMD_for	ATGgaagtctgcaattgtattgaaccg
ETR1-TMD_rev	taaagtgcttctaatctcatgagtcaacattctc
